# Handling and Physiological Aspects of the Dual-Purpose Water Buffalo Production System in the Mexican Humid Tropics

**DOI:** 10.3390/ani12050608

**Published:** 2022-02-28

**Authors:** Daniela Rodríguez-González, Antonio Humberto Hamad Minervino, Agustín Orihuela, Aldo Bertoni, Diego Armando Morales-Canela, Adolfo Álvarez-Macías, Nancy José-Pérez, Adriana Domínguez-Oliva, Daniel Mota-Rojas

**Affiliations:** 1 Master’s Program in Agricultural and Livestock Sciences [Maestría en Ciencias Agropecuarias], Xochimilco Campus, Universidad Autónoma Metropolitana (UAM), Mexico City 04960, Mexico; drg.mvz02@gmail.com; 2 Laboratory of Animal Health, LARSANA, Federal University of Western Pará, UFOPA, Rua Vera Paz, s/n, Santarém 68040-255, PA, Brazil; 3 Facultad de Ciencias Agropecuarias, Universidad Autónoma del Estado de Morelos, Cuernavaca 62209, Mexico; aorihuela@uaem.mx; 4 Neurophysiology, Behavior, and Animal Welfare Assessment, Department of Animal Production and Agriculture (DPAA), Xochimilco Campus, Universidad Autónoma Metropolitana (UAM), Mexico City 04960, Mexico; aldo_bm@hotmail.com (A.B.); damoralesc@outlook.com (D.A.M.-C.); aalvarez@correo.xoc.uam.mx (A.Á.-M.); nancy.jopz@gmail.com (N.J.-P.); mvz.freena@gmail.com (A.D.-O.); 5 Holistic Management, Savory Institute, Agriculture Science, Universidad EARTH, San José 4442-1000, Costa Rica

**Keywords:** allosuckling, *Bubalus bubalis*, calving, imprinting, Mexico wetlands

## Abstract

**Simple Summary:**

Buffalo is a domesticated large ruminant that can be raised for beef, dairy, and work. In some systems, these animals can be raised with a dual purpose (beef and dairy). The present review describes the characteristics of the dual-purpose water buffalo production system in Mexico’s humid wetlands. This article provides extensive information on the water buffalo and includes comparisons with other species to note similarities and differences. The aim is to describe the buffalo handling procedures used in this system, particularly during breeding, milking, confinement, and mobilization, relating them to the neurological processes involved and analyzing the productive results. Understanding these processes will allow us to obtain a more precise vision of the advantages that this species can offer, and the possible implications of the development of this type of livestock under tropical conditions.

**Abstract:**

The purpose of this paper is to describe the characteristics of the dual-purpose water buffalo production based on the Mexican production system as a model in tropical wetlands. It includes a broad literature review emphasizing the most recent and specialized publications examining key findings to improve our understanding in the performance of the buffalo species (*Bubalus bubalis*). The complementary topics addressed include reproductive management, parturition, the dam–calf bond, milking routines, and models of confinement and management, in addition to aspects related to milk commercialization. This article summarizes the advances made to date in this production system and its current margins for improvement. The development of dual-purpose water buffalo production systems in Mexico’s tropical wetlands is a relatively recent phenomenon that has progressed and improved due to herd management. Buffaloes are an interesting alternative for dual purpose systems that offer several advantages. The lower milk production of this species compared to cattle is its main limitation. However, the properties of their milk allow one to obtain an added value and make this type of farms competitive. In synthesis, consolidating buffalo production in Mexico’s tropical wetlands will require broadening our knowledge of this species, and perfecting the most appropriate handling procedures. The activities of government agencies and processing enterprises will play vital roles in achieving the integral modernization of this potentially important economic activity.

## 1. Introduction

In recent years, the FAO has maintained that one of the principal challenges facing global society is alimentary insecurity, given the high levels of poverty and the difficulties that existing agricultural and food systems encounter in guaranteeing food supplies and their adequate distribution [1]. The FAO estimates that by the year 2050, the world’s population will be 9.73 billion, which means that food production must increase by 49%. It also stresses that this is not just a future problem, but also a current one, as about two billion people have micronutrient deficiencies, and almost 800 million suffer from chronic hunger.

In response to global food insecurity, the expansion of water buffalo could be considered because they can thrive in many regions with low agricultural potential. Despite recent controversies surrounding the raising of large ruminants, the physiological and technical characteristics of the water buffalo have stimulated the growth of its populations worldwide. This has been achieved specifically due to its rustic nature and productive efficiency in regions with low agricultural potential, such as wetlands, areas of difficult access, and regions with medium- and low-quality grasses. In these kind of habitats, the adequate management of this species can contribute to a process of progressive regeneration of pasture [1,2,3,4,5].

The water buffalo is ranked as the sixth most abundant productive species on the planet, surpassed only by inventories of poultry, conventional bovines (genus *Bos*), swine, sheep, and goats [6]. In 2018, the FAO reported a population of 206 million water buffaloes worldwide, distributed in 48 countries, concentrated mainly in Asia, with roughly 97% of the total population, especially India, China, and Pakistan. The remaining water buffalo are distributed Africa (1.7%, mostly in Egypt, the Americas (1.2%), Europe (0.2%), and a small herd in Oceania (0.07%) [2]. Other important data presented by the FAO include the fact that the growth of water buffalo populations around the world in the 2008–2018 period was 11%, 6% greater than that of *Bos* cattle in the same period [1].

Unverified reports on Mexico estimate a water buffalo population of 45,000 individuals, distributed primarily in tropical wetland areas where a dual-purpose buffalo production system (DPBPS) has been adopted, obtaining meat and dairy products simultaneously. To date, only a few documents have characterized these enterprises in the tropical areas [7,8,9,10]. The equal priority that female and male animals require in dual-purpose production units requires complementary forms of management, especially for processes such as breeding.

Considering the above, the aim of this review article is to describe and characterize the key technical and commercial processes and practices of dual-purpose water buffalo production systems in Mexico’s tropical wetlands, with an emphasis on reproductive, physiological, and behavioral aspects, among others, that are strategic for the efficient development of these systems. The broader objective is to advance towards the goal of achieving efficient, sustainable production models.

## 2. Materials and Methods

This study was based on a broad literature review that focused on the most recent publications examining information that contributes to a better understanding of the performance of this animal species.

**Figure 1:**
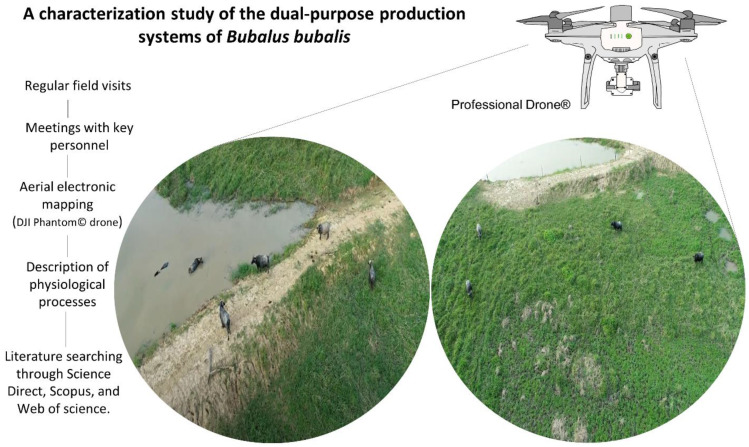
Methodology to obtain the data and describe the dynamic process in the dual-purpose production system of water buffaloes (17°38′54.0″ N 94°36′05.0″ W). Signs of estrus and parturition are facilitated by this method. The data obtained through regular field visits and aerial mapping with a DJI Phantom 4 Pro V2.0, DJI, Shenzhen, China, drone were compared to recently published scientific references.

To visualize and analyze the processes employed on water buffalo ranches, we made periodic visits to production units and programmed meetings with key actors, including owners and administrators, coupled with an update of the scientific literature available. The visits were performed for three months, starting in August, and ending in October 2021; from 04:30 h to 19:00 h. We utilized technological tools to map study areas by means of infrared thermographic evaluation and the observation of the operations conducted. To assess such topics as production flows, pasturing zones, the mobilization and transport of animals, and how handlers identify sick animals or females during calving, a drone was flown daily over the study area (DJI Phantom Drone 4 pro V2.0, DJI, Shenzhen, China) at an altitude of up to 60 m (Figure 1). Regarding the thermographic images, an infrared camera was used (FLIR Thermal TM E95, FLIR Systems Inc., Wilsonville, OR, USA) with an emissivity of 0.95 and IR resolution 464 × 348. Our characterizations of the production processes in each zone include descriptions of important physiological and neurobiological processes of the water buffalo during the reproductive phase of the estrus and the use of artificial insemination, and explaining the neurobiological processes involved in calving, imprinting, milking, and allonursing. Our goal was to obtain a broad panorama of the physiological particularities of female buffaloes as a species and, where possible, identify aspects that require improvement. Finally, we elaborated a map of routine processes and elaborated a descriptive discussion of neurobiological processes. These activities required a broad bibliographic review and consulting and selecting scientific articles available in several databases, including ScienceDirect, Web of Science, Scopus, and PubMed, using the following keywords: “water buffalo”, “river buffalo”, “domestic buffalo”, “breeding”, “dystocia”, “parturition”, “productivity management”. The information chosen for analysis was recent and, preferably, of high impact.

## 3. Reproductive Management of Female Buffaloes

### 3.1. Genetic Selection

**Figure 2:**
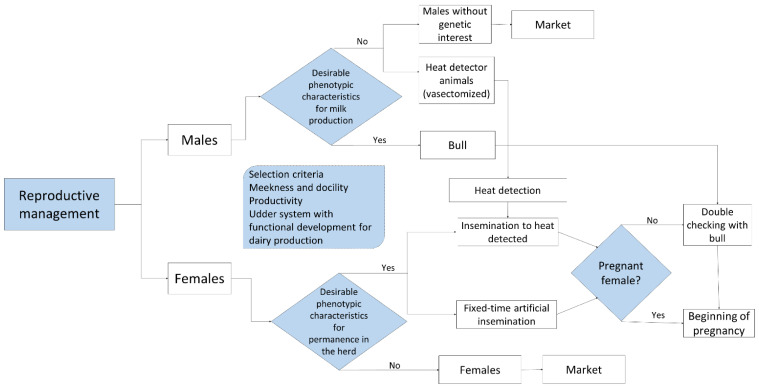
Reproductive management in the dual-purpose production system of water buffalo housed in the humid Mexican tropics.

The genetic selection of water buffaloes is based on traditional criteria such as levels of milk production and docility. Animal docility, milk production, and maternal ability traits are phenotypic characteristics selected in the farms by producers. This means that both qualitative and quantitative characteristics are considered in efforts to optimize the entire system, and this process entails evaluating traits associated with high productivity, such as an adequate body structure that permits ingesting and then transforming food to build muscle, and the development of a mammary system capable of synthetizing large amounts of milk [10,11]. Another feature commonly assessed is the conformation of the legs and hooves, as they play an essential role in buffalo health [12], allowing them to move through pasturelands for forage access. In synthesis, these varied selection criteria all play key roles in the level of productivity of dual-purpose systems in genetic and reproductive management. In the case of males used as stallions, the selected animals must be from artificial insemination with a birth weight above the average, and should have desirable behavioral aspects such as docility and meekness. In females, these behavioral characteristics are also considered, in addition to a record of the parents and phenotypic characteristics such as mammary gland development and kilograms of milk produced by lactation, information retrieved from production records (Figure 2).

Breeders consider essential selection criteria in efforts to improve the selection of dams and bulls on their ranches. The latter are selected from catalogues that stress the genetic value that they can pass on to their descendants. This genetic material is normally utilized through artificial insemination, in some cases when estrus is detected, but more often at fixed times. Studies have detected that the Buffalypso breed is the one most often utilized in DPBPS, although breeds with greater milk-producing potential, such as Mediterranean Italian and Murrah, are being introduced gradually. This has entailed implementing assisted reproduction technologies, as we describe in the following section [8,13,14].

### 3.2. Estrous Detection

**Figure 3:**
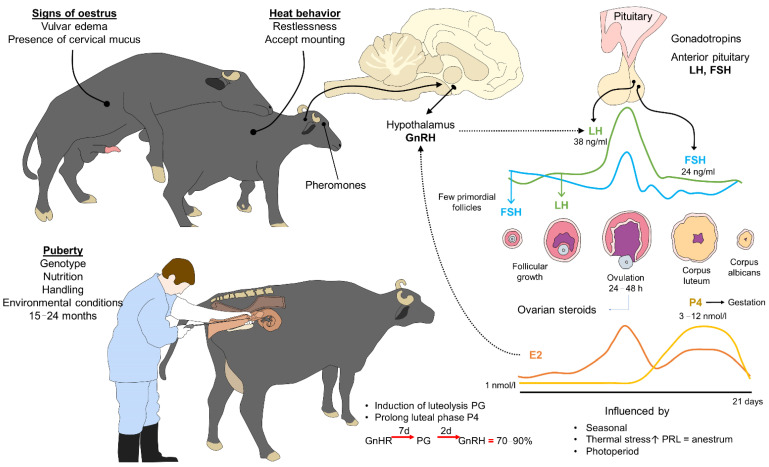
Neuroendocrinology of the estrus cycle, estrous detection, and artificial insemination technique. During estrus, multiple signs and behaviors may be present in female buffaloes. Concentrations of LH and FSH, two gonadotropins secreted by the anterior pituitary through the action of the hypothalamus, are the main hormones involved. When LH and FSH levels are sufficiently high, ovulation takes place (lasting 24–48 h in buffaloes) with the consequent secretion of E2. Estrogen levels decrease gradually while progesterone levels increase to restart the estrous cycle. Heat detection based on signs, behaviors, or hormone levels is an essential aspect of artificial insemination programs, where one main goal is to avoid influences by season or other factors. E2: 17β-estradiol; FSH: follicle-stimulating hormone; GnRH: gonadotropin releasing hormone; LH: luteinizing hormone; P4: progesterone; PG: prostaglandins; PRL: prolactin.

Identifying estrus in female buffaloes is especially complicated because these animals manifest the signs commonly seen in cows including vulvar edema, frequent urination, and vaginal secretions with very low intensity [13]. For this reason, breeders have opted to use a vasectomized male to identify females that are viable for insemination. Female buffaloes have a small follicular size that generates low concentrations of estradiol. Research has documented that 3.4% of female buffaloes manifest estrous behavior and that over 60% have what is called “silent estrus” [8,15]; thus, some ranches have implemented fixed-time reproductive protocols that foster genetic improvement by responding to the physiological characteristics of female buffaloes if no male capable of identifying estrus is available (Figure 3). In addition to this, non-invasive tools such as infrared thermography have been used in the detection of estrus due to the changes presented in the vulva prior to ovulation, which could be an efficient and safe indicator of estrus [16,17,18,19].

### 3.3. Artificial Insemination

Artificial insemination has been widely incorporated into buffalo production systems in Mexican tropics, so the females selected to remain with the group and that are viable for reproduction are subjected to methods of assisted reproduction. Once a vasectomized male identifies the females that display natural estrus, they are inseminated artificially at a fixed time, while the others are included in a protocol that consists of applying a hormonal treatment prior to insemination. In both cases, the females that do not become pregnant are served directly by a bull to improve pregnancy rates. This makes it possible to scale the birth of calves throughout the year, keeping milk production relatively stable [14]. Thus, we believe that the joint implementation of reproductive biotechnologies facilitates advancing genetically towards the goals of achieving productive transcendence and maintaining controlled estrus cycles (Figure 3). Other reproductive technologies, such as embryo transfer (ET) or sexed semen, are not utilized; the first due to sanitary restrictions on their importation, and the second because of high costs.

## 4. Calving

Calving begins physiologically at least ten days prior to parturition, which occurs after 300–329 days of gestation (mean range of 310–315 days) [20,21,22]. For calving, cows are taken to a secure area for continuous supervision and, if necessary, timely obstetric intervention. Once installed in the maternity paddock, the female buffaloes are evaluated daily to ensure that calving is detected promptly. The personnel in charge verify the condition of the udders, the frequency of urination, and postural changes such as repeatedly lying down and standing up, all of which indicate that calving is imminent [23].

The area set aside for calving and the onset of maternity must have certain, basic physical and biological characteristics, including native forage such as Camalote (*Paspalum fasciculatum*) and Azuche (*Hymenachne amplexicaulis*), which are the primary forages for this species in the region [24,25]. The dams’ daily diet is often supplemented with 50 g of minerals [26]. This area must also have tree species that provide the shade that female buffaloes require for thermoregulation [27]. Other recommendations in the calving area are to supply drinking water in troughs and avoid deep bodies of water to eliminate the possibility of drownings. When the cow is about to give birth, monitoring can be complemented using a drone, a technological tool that does not interfere with the animals or cause the stress that can arise from human presence. The objective is to identify any cow that presents symptoms of dystocia during parturition and immediately call for veterinary attention, though this is rarely required [28].

When brought to a satisfactory conclusion, parturition is considered successful thanks, in part, to the constant supervision of the females that are close to delivery. Although the frequency of dystocia is low in buffaloes, it may occur [29]. This condition not only threatens the welfare of both dam and offspring, but also means economic losses for producers, especially if they have invested significant economic resources in implementing reproductive protocols such as artificial insemination.

The following section describes the calving process from a physiological perspective, including both normal (eutocic) deliveries and deviations that can occur. The latter are considered dystocic deliveries and require veterinary assistance.

### 4.1. Eutocic Births

According to the literature, labor in buffaloes begins with the onset of regular uterine contractions accompanied by the progressive dilatation of the uterine neck. This typically occurs in three stages: dilatation of the cervix, expulsion of the fetus, and expulsion of the fetal membranes [23,28,30].

The first stage usually lasts 1–2 h, possibly longer in primiparous females [31]. In this stage, the buffaloes show a structural change in the dilatation of the uterine neck, triggering the onset of contractions of the myometrium. Then, the fetus adopts the position for expulsion [32]. The cow may seem restless and her heart rate and respiratory frequency may increase [30,32].

Ensuring the availability of natural shade in the birthing area where the prepartum female buffaloes are held could have a positive effect on the parturition process, reducing the incidence of thermal stress and the probability of suffering placental hormonal imbalances that could negatively affect the birth weight of her calf and interfere with the passive immunity acquired by the calf when ingesting colostrum [33,34]. Mudgal [35] suggested that supplementing the diet of multiparous female Murrah buffaloes with vitamins A and E had positive effects on increasing the protein and total solid content of their colostrum during the first 2 to 3 d postpartum, respectively, compared to a group of females that did not receive supplementation.

**Figure 4:**
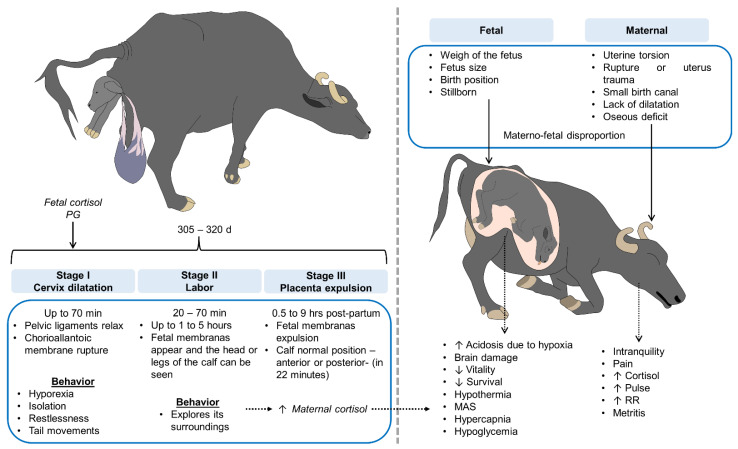
Stages of parturition and maternal and fetal causes of dystocia in the water buffalo. MAS: meconium aspiration syndrome; PGF2 alfa: prostaglandin F2 alpha; RR: respiratory rate.

It is important to underline the importance of electronically monitoring the females during calving with a drone to detect signs of the approaching delivery. Telltale signs include separation from the group, a continuous standing posture, frequent tail movements and vocalizations. In addition, recent studies have documented other observable signs, such as sagging of the sacrosciatic and sacroiliac ligaments 12–24 and 24–72 h prepartum, respectively. The relaxation of these pelvic ligaments results in an apparent raising of the base of the tail. In some cases, it might be possible to observe edematization of the vulva with the presence of crystalline mucus around 72–96 h prepartum. In addition, 24–36 h before calving, the vulva appears extremely flaccid and the cow’s teats look distended. Then, 1.8 d before delivery, the mammary veins appear tense [36]. Additional signs may appear because of pain. These include nervousness, reduced appetite, and postural changes (lying down/standing up and vice versa). Cows also tend to look at their flanks constantly, keep their heads raised, scratch the ground, and raise their tails during contractions. Handlers may also observe lateral tail movement, arching of the back, and flexions of the hocks accompanied by restlessness [36] (Figure 4). Watery diarrhea has been reported as another sign of approaching delivery [36].

The second stage, expulsion of the fetus, is marked by strong uterine and abdominal contractions and cervical dilatation [30]. Other signs are the rupture of the allantochorion, the release of liquid through the vulva, the appearance of the amnion in the vulva, its breakage and, finally, the expulsion of the calf [30]. This stage generally lasts 30–60 min [31].

The third phase occurs when the dam expels the fetal membranes. It usually lasts 4–5 h after the expulsion of the calf [31]. During this stage, the intensity of the uterine contractions diminishes, lessening the dam’s physical exertions and paving the way for the shedding of the chorionic villosities from the maternal crypts [30,32], Finally, placentophagia occurs, an observed behavior that consists of the consumption of placental components by the buffalo [37].

A study by Deka et al. [38] evaluated the behavior of water buffaloes during the three stages of calving. They determined that in the first stage, 100% of females showed nervousness, reduced food and water consumption, displayed postural changes (standing or in repose), tail-raising, abdominal exertion, arching of the back, vaginal secretion, and frequent urination. For stage 2, they found that the buffaloes showed signs akin to those observed in stage 1, except for tail movements (in 100% of cases). The behavior of the buffaloes in stage 3 was characterized mainly by licking the neonate and renewed interest in food and water, with occasional manifestations of abdominal effort [38].

### 4.2. Dystocic Calving

The importance of understanding the three stages of parturition in female buffalo, including the associated times, behaviors, and signs, lies in recognizing when labor is prolonged, ensuring that the cow expels the fetus in a normal position, or opportunely determining when some unexpected event occurs that could indicate an anomaly in the calving process. Dystocia has severe consequences for both dam and fetus (neonate) that may include uterine infections, placental retention, excessive expulsion time, decreased milk production and, in extreme cases, increased mortality and morbidity of dam and calf [39].

The occurrence of dystocic births in DPBPS is uncommon. In the isolated cases that do occur, handlers are limited to help the female by means of manual traction manipulations to aid in expelling the fetus. Surgical techniques and pharmacological therapies are not routine obstetric practices because veterinarians are rarely called in to assist in deliveries involving female buffaloes.

In contrast to cattle (*Bos taurus* and *B. indicus*), female buffaloes show a low predisposition towards dystocic births due to key anatomical differences, such as their pelvic and genital structure. The iliac area of buffaloes is larger than in cattle, and the fifth sacral vertebra is separated, allowing greater freedom of movement and a wider birth canal that facilitates expulsion of the fetus [40,41]. The morphology of the female buffalo’s reproductive tract includes longer, wider vaginal lips, and a small vaginal canal that dilatates easily to help complete labor in a shorter time than in cattle [40]. For example, some studies have documented that 5% of the total of dystocic births attended in cows are attributed to their narrow pelvic canal [42], in sharp contrast to the low percentage reported in buffaloes (1.6%) (Figure 3). The pelvic measure is used to assess in cattle if the canal is narrow. The pelvic area obtained before breeding divided by the estimated calf birth weight is considered a reliable indicator of dystocia [43].

Although the DPBPS reports low incidences of dystocic births in female buffaloes in Mexico’s tropical wetlands, retrospective studies in India—the principal water buffalo-producing country in the world—reported the incidence of such births in buffaloes at 80.3%, which is a similar rate to what has been observed in cattle (78.89%) [42]. This could be due to different obstetric management in Asia, or a different genetic line that could predispose buffalo females to developing dystocia, such as a weaker broad ligament, a factor that can be found in newly born buffalo calves [44]

In mammals in general, complications during birth are attributed to either maternal or fetal causes. These conditions are examined separately in the following sections.

#### 4.2.1. Maternal

Maternal conditions, such as uterine torsion, narrowing of the birth canal, and incomplete cervical dilatation are the main causes of complications in female buffaloes [45]. Numerous studies have reported that approximately 53.6% of dystocic births in female buffaloes can be attributed to such maternal factors [40,46]. In one experiment with 142 Murrah buffaloes, 59.2% of the dystocic births were due to maternal causes, with the main etiology being uterine torsion (83.3%) [47]. These findings coincide with the results of a study by Jeengar et al. [42], in which for 55.7% of the 51 buffaloes that had dystocic births, dystocia was caused by uterine torsion. While figures for the survival of the dams in those cases were high (90%), up to 70% of the newborns died as a result of the dam’s condition [48]. Clearly, this is a topic that water buffalo breeders must address.

Uterine torsion occurs when the uterus twists (90–120 degrees) along its longitudinal axis. The point of torsion is classified as post- or pre-cervical, with the former being more frequent (98.4%) [49]. Most of the females that present this condition do so during the final stage of gestation (5–8 mo) [50]. Uterine torsion occurs more often in multiparous than primiparous females (81.7 vs. 18.3) [49]. Some of the signs associated with this pathology are hyperoxia, constipation, colic, and straining [48]. One reason why uterine torsion is the main etiology of dystocic births in female buffaloes has to do with the anatomy of *Bubalus bubalis* dams. These animals have a wide, but weak, ligament with few, underdeveloped, muscle fibers [51]. Because this wide ligament is not fixed to the dorsal or right walls and lacks muscle folds, the ligament is prone to twisting [50]. Regarding this condition, a study of 20 buffaloes by Ali et al. [49] estimated that 96% of uterine torsions were towards the right (clockwise), due to the characteristics of this ligament compared to the one on the left side where the rumen is located [50]. Other elements associated with uterine torsion are low amounts of fetal fluids, reduced tone and size of the uterus at the end of gestation that increase fetal movement, cases in which the uterus is not anchored appropriately and so can twist on its own axis, the female buffalo’s pendulous abdomen, and wallowing behavior, though the role of the latter factor has not yet been objectively demonstrated [50].

Maternal causes of dystocia, especially uterine torsion, have numerous effects that run from a high incidence of stillbirths to organic imbalances and alterations in the dam [23,28]. In this vein, in a study of 28 female buffaloes that presented dystocia, their biochemical and mineral profiles showed significant increases in plasma iron, copper, zinc, calcium, aspartate aminotransferase, alanine aminotransferase and alkaline phosphatase concentrations [52]. Likewise, hematological parameters such as high levels of urea, nitrogen, and phosphorus, accompanied by decreased mean corpuscular hemoglobin, derive from insufficient irrigation that can affect renal and liver function [49]. Creatinine, progesterone, cortisol, tumor necrosis factor (TNF-α), interleukin 6 (IL-6), and glucose concentrations also increase in response to the stress caused by prolonged births and pain [48]. Research on dystocic female buffaloes has verified that plasma cortisol concentrations may almost double in animals with dystocic vs. eutocic births (64.3 +/− 10.1 ng/mL vs. 39.3 +/− 2.0 ng/mL, respectively) [53], and that this can have lethal consequences for dams and offspring.

Other consequences are adherence to abdominal tissues, edema, intense uterine contractions, increases in creatine phosphokinase (CK), and placental retention [50]. If the fetal membranes have not been expelled after 12 h (normal time = 0.5–8 h postpartum), there is a risk that the dam may develop metritis, endometritis, or pyometra [28].

#### 4.2.2. Fetal Factors

One of the most common causes of dystocic births is maternal–fetal disproportion [41]. Srinivas et al. [47] reported that 40.8% of dystocic births in female Murrah buffaloes are due to fetal factors, including oversized fetuses and abnormalities such as deformities, congenital and acquired diseases (e.g., hydrocephaly, ascites, anasarca, hydrothorax), and inadequate positioning in the birth canal. On the topic of fetal size, Kumar et al. [54] reported a dystocia incidence of 40.8%, and found that 22.4% of these cases were due to an oversized fetus, a condition that requires fetotomy (if the fetus is dead inside the uterus) or caesarean section. In some cases, a size disproportion can cause pathologies or deformities in the fetus. For example, though rare, fetal ascites—due to insufficient drainage of the peritoneal fluid or reduced excretion of urine [41]—and congenital diseases such as hydrocephaly (present in India in 1.5 of every 1000 births) increase the size of the fetus’ body or head, preventing it from passing through the birth canal [55]. A similar phenomenon has been detected in cases of deformities (monstrous births). Though the latter events are extremely rare, several cases have been identified by Gahlod et al. [56] and by the *Instituto de Educación e Investigación Veterinaria* [57].

The position that the calf adopts during parturition has a significant influence on whether the birth will be eutocic or dystocic. Dystocic births occurred mostly with an anterior (86.7% of cases) presentation rather than with a posterior (13.3%) one; deviated limbs (57.8%) were more frequent than deviations of the head (42.2%), regardless of the dystocic presentation [47]. Severe lateral torsions of the head and neck carry a high risk of fetal mortality [58]. Abnormal positions and difficult expulsions affect both the newborn and dam, especially in production units where births are overseen.

Intervention during a dystocic birth consists of manually pulling the fetus out of the birth canal; but if this movement is not executed with great care, or if an incorrect technique is used, severe perineal lesions may occur, including infection and necrotization, and surgical intervention might even be required [59].

Consequences for the neonate depend largely on the time between what began as a normal birth and the identification of a condition with dystocic features. The longer this period is, the more severe the repercussions for the calf’s health are. Studies have documented that the second stage of a eutocic birth lasts around 20–70 min. When this time is exceeded, the risk of fetal acidosis (caused by hypoxia or anoxia) increases. This condition can trigger organ failure and even death of the newborn [60]. Other problems identified are hypercapnia, hypothermia, hypoglycemia, meconium aspiration syndrome, and ruptured umbilical cord. All these factors can predispose calves to other pathologies or low vitality during the first hours of life [28]. In general, dystocic births, whatever their cause, are accompanied by biochemical, physiological, and behavioral alterations, such as restlessness, pawing the floor, or arching the back due to pain [61]. The importance of monitoring parturitions ensures the early detection of abnormalities during labor, so that strategies can be implemented immediately to prevent more severe consequences for both dam and calf.

## 5. Dam–Offspring Bond

**Figure 5:**
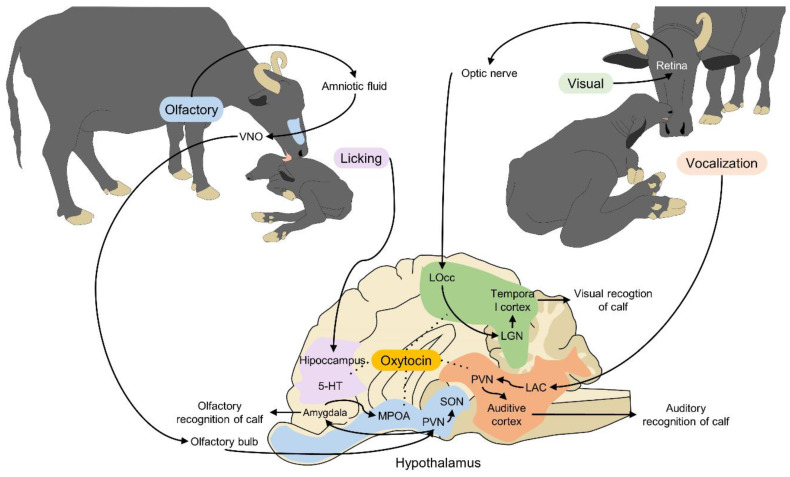
Olfactory, auditory, visual, and tactile recognition of calves in the water buffalo (*Bubalus bubalis*). Various structures and brain nuclei are activated when a calf is born. First, the amniotic fluid activates the VNO. The VNO, through the olfactory bulb, projects the stimuli to the PVN, SON, MPOA, and amygdala. Licking the fetal membranes off the newborn activates areas of the hypothalamus that release 5-HT and oxytocin. Visual recognition of the calf is achieved through the projection of the optic nerve to the Locc, LGN, and temporal cortex. Vocalizations contribute to recognition with the LAC, PVN, and auditory cortex all playing important roles. All these stimuli participate in the secretion of oxytocin, the main neurotransmitter involved in maternal behavior. 5-HT: serotonin; LAC: left auditory cortex; LGN: lateral geniculate nucleus; Locc: occipital lobe; MPOA: medial preoptic area; OXT: oxytocin; PVN: paraventricular nucleus; SON: supraoptic nucleus: VMH: ventromedial nucleus of the hypothalamus; VNO: vomeronasal organ.

During the expulsion of the fetus, handlers in the calving area generally watch over movements of the limbs and the thoracic region to verify adequate respiration. If a lack of movement is detected in the calf, the observers must enter the paddocks and approach carefully to determine if it requires assistance or, in the worst case, is stillborn. Observers should also ensure that the dam stays close to the calf and begins calf–cow interaction by licking the offspring to remove the fetal membranes. If the dam moves away from a calf that is still unable to stand or suckle, handlers must attempt to facilitate the formation of the bond between the dam and the newborn. If the dam continues to reject her offspring, the neonate will need to be integrated into a process of communal rearing where it will be fed by surrogates, a method that has been seen to be highly feasible among water buffaloes. Keeping the dam and calf together, followed by the onset of suckling—including colostrum ingestion—are especially important processes that are intimately related to the survival and development of these animals (Figure 5).

### Neurophysiological Mechanisms between Dam and Offspring Activated by Sight, Licking, Smell, and Vocalizations

At birth, the dam is the calf’s first social contact and main source of learning. At her side, the newborn memorizes the behavioral patterns necessary for its survival and other important information concerning its physical and social environment [62]. Establishing the dam–calf bond after parturition is, therefore, a transcendental step, especially in production systems based on free pasturing [63] due to the risk of losing those calves in extensive pasturelands [64,65]. The formation of the dam–calf bond involves learning processes that permit a clear acquisition of information and the establishment of preferences through specific sensory stimuli (sight, touch, hearing, smell, taste) [62]. In the case of buffaloes, sight and smell are the most active senses [65], as they consist of a series of sensory communication channels that consolidate mutual recognition and have a lasting influence that resists eventual temporary separations [64] (Figure 5).

During stage 2 of parturition, imprinting occurs, which starts when the product begins its passage through the cervical-vaginal portion of the birth canal [23]. Stimulated by the transit of the fetus, mechanoreceptors in the uterus send information through the spinal cord to hypothalamic structures as the paraventricular and supraoptic nuclei, which stimulate the release of oxytocin from the posterior hypophysis [65]. Oxytocin’s dual action generates simultaneous responses, generating contractions of the birth canal, and acting as a neurotransmitter in the dam’s olfactory bulb that aids in the secretion of dopamine, which triggers the process of mutual recognition between dam and calf [64].

In water buffaloes, the most common pattern of maternal behavior in the first 6 h after birth is licking [66]. Around this time, the calf makes its first attempts to stand up, seek the udder, and suckle to receive the nutritional and immunological benefits that the dam’s milk provides. The dam facilitates access to her teats by arching her back and flexing her hind limbs. Allosuckling is often seen in multiparous female buffaloes, accepting calves from different biological dams. This behavior, considered altruistic, is examined in detail in subsequent sections.

Calves and dams can perceive a series of signals that respond to auditory, visual, olfactory, and tactile stimuli. The olfactory pathway is thought to be the most direct one between the environment and brain, and actively participates during imprinting. As with taste, smell is classified as a chemical sense that has the capacity to capture volatile and non-volatile chemical substances by means of receptors coupled with G proteins [67]. The sense of smell is integrated with two pathways: the main olfactory epithelium and the vomeronasal organ (VNO). The first detects odorific chemical substances suspended in the air, associated with functions such as alimentation and predator detection. The second identifies non-volatile chemical substances, such pheromones, more commonly related to sexual behavior [65,67]. Both pathways convey signals to the olfactory bulb, which evaluates the odorific configuration detected and sends signals to the aforementioned hypothalamic structures—paraventricular (PVN) and supraoptic nuclei (SON)—which in turn contain neuroendocrine cells that synthetize oxytocin [62]. These signals also reach limbic system structures. The hippocampus stores the information, and the medial portion of the amygdala (MPOA) generates emotional responses linked to the stimuli, including maternal recognition, attachment to the calf, and reproductive functions [64].

Visual imprinting begins with preliminary recognition which occurs thanks to the newborn calf’s ability to follow a moving object or being that offers protection and sustenance. Visual stimuli are projected through the optic nerve to the occipital lobe (LOcc). The lateral geniculate nucleus (LGN) receives the visual information perceived and sends the signal over the geniculate-striatal pathway to the visual cortex, which decodifies it and transforms it into visual characteristics [64]. Normally, the definitive recognition of calves occurs through olfactory stimuli, with visual communication playing a complementary role [65].

The auditory communication pathway permits acoustic recognition that stimulates the care of the offspring based on the emission and recognition of auditory patterns. This pathway operates bidirectionally between the dam and calf: on one side, an emitter that conveys differentiated vocalizations, and on the other, a receptor that captures clearly recognizable auditory patterns. This link can also transcend temporary separations [65]. The vocalizations emitted are projected to the auditory thalamus, which conveys the signals to the PVN and auditory cortex where the bidirectional auditory recognition of the emission takes place [64].

All the communication pathways that generate and maintain the dam–calf bond are related to structures that release oxytocin and positive (satisfactory) emotional responses. The oxytocinergic and dopaminergic systems are the biological systems involved in maternal recognition and care. Of prime importance here is the release of oxytocin to activate dopamine pathways [65].

## 6. Colostrum

### Colostrum Ingestion in the Dual-Purpose System

The mixture of milk secretions, immunoglobulins, and other serum proteins that accumulate in the dam’s mammary gland during the prepartum period are defined as colostrum [68]. There is a consensus that the adequate management of colostrum plays a key role in determining neonatal health and survival [69]. Compared to cow colostrum, buffalo colostrum contains higher concentrations of fat (8.04% vs. 9.59%), lactose, ash, vitamin E (234 IU/100 mL vs. 342 IU/100 mL), total solids (24.19% vs. 26.67%), phosphorus (53 vs. 58 mg/100 g), and IGF-1 [70]. Analyses of the colostrum of female Murrah buffaloes have determined that the predominant white blood cells are macrophages, followed by lymphocytes and neutrophils. Phagocytic activity at birth was measured at 23%, but decreased drastically to 14% on day 5 postpartum [71].

**Figure 6:**
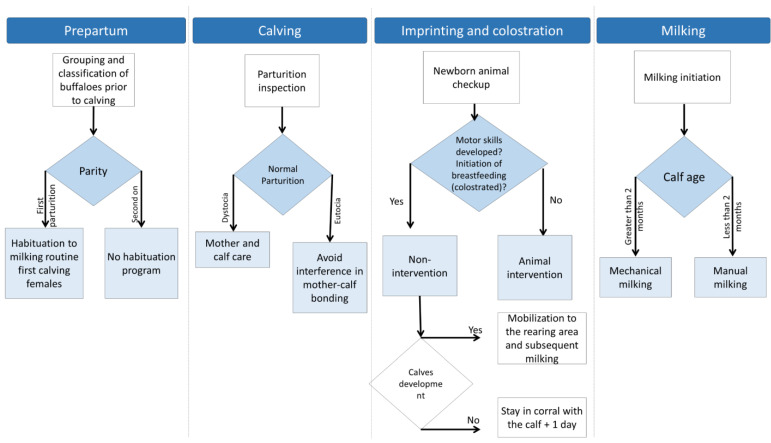
Flow diagram of the prepartum, calving, imprinting, colostrum, and milking processes in the dual-purpose buffalo production system in Mexico’s tropical wetlands.

During the development of the dam–calf bond in production units in the tropical wetlands of Mexico, the dam and its calf remain together for one day, to permit the calf to suckle for the first time. Subsequently, both are guided to the maternity area, where they stay for an average of 12 days, considering the adequate motor development of the calf. After this period, the female is incorporated into the milking process, and the calf is used to stimulate milk ejection during the milking (Figure 6).

This process generates two transcendental effects. First, it allows a high number of calves to benefit by ingesting colostrum from different dams through allosuckling. Second, it favors the conditioning of the cow for the milking period, whether manual or mechanical. Colostrum ingestion is important because it confers passive immunity by transferring antibodies and leucocytes of maternal origin [72,73] to the calf during the first hours post-birth. It is important to consider that the absorption of immunoglobulins ends within 36 h postpartum. During this interval, only the abomasum is active, so colostrum ingestion not only exerts effects on the calf’s immune system but also supplies valuable nutritional compounds, such as vitamins, fats, and proteins [72,74]. The practice of allonursing among water buffaloes also has two main repercussions; it can compensate for any nutritional deficiency in the calves, but it might increase the risk of the transmission of pathogens [62].

What is clear, despite this potential risk, is that allonursing favors greater colostrum ingestion during the first days of life, which is important in light of the reduction in fats, proteins, and dry substance [74], as well as in antioxidant parameters (Trolox equivalent antioxidant capacity, total phenolics, reducing power, and DPPH radical scavenging activity) that occurs postpartum [75]. This has been demonstrated by analyses of colostrum collected from female Murrah buffaloes on days 1 to 5 postpartum, which show that levels of the immunoglobulins IgA and IgM (3.22 mg/mL and 5.22 mg/mL, respectively) decrease during these 5 d. Moreover, somatic cell counts are elevated (500,000/mL) at the moment of birth, but diminish markedly by day 2 and then more gradually up to day 5 (180,000/mL) [71].

**Figure 7:**
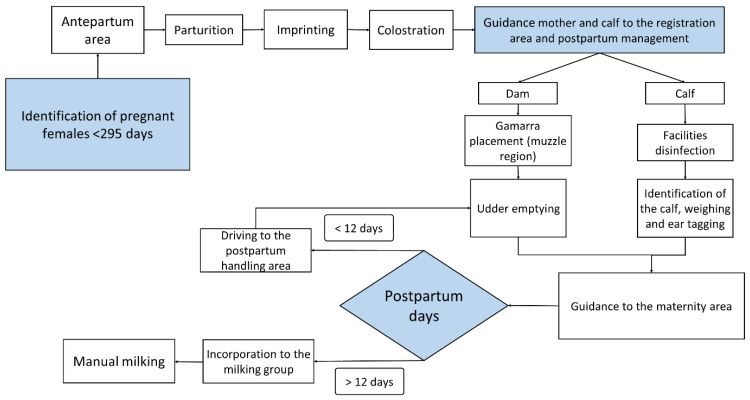
Criteria for handling in the calf and dam.

It is important to emphasize that the simple act of allowing calves to ingest their dams’ milk for a period of 90 days (one month more than is normally allowed in production units in Mexico’s tropical wetlands) positively impacts weight gain (0.506 vs. 0.438 kg/day). In addition, allowing calves to suckle reduces the time spent on crossed suckling and licking themselves and other objects, compared to calves that are weaned immediately after birth [76] (Figure 7).

## 7. Milking Handling

**Figure 8:**
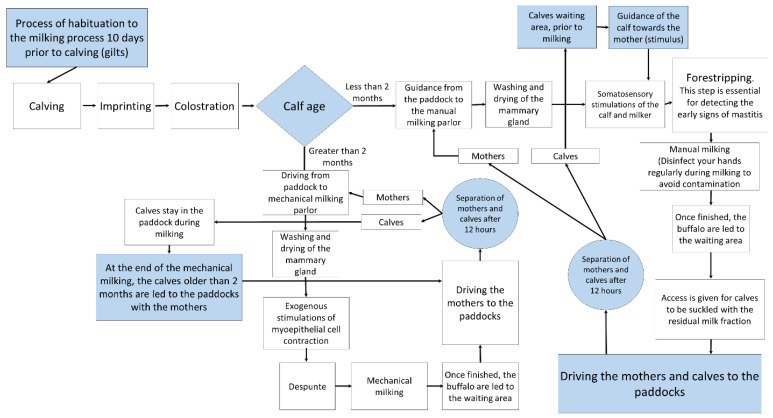
Flow chart of the characteristic decisions required in the manual and mechanical milking processes of water buffaloes.

**Figure 9:**
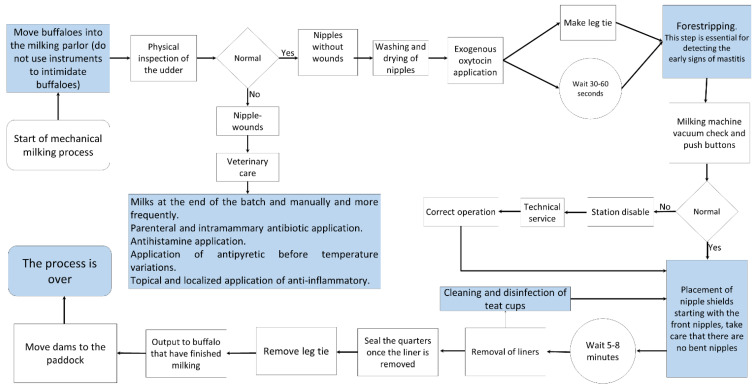
Flow chart of the characteristics of the mechanical milking process. In the dual-purpose production system, the presence of clinical or subclinical mastitis is low; however, when it occurs, veterinary attention is necessary.

Lactation in female buffaloes in the study area begins shortly after birth and lasts an average of 240 days, but it is largely influenced by several factors such as breed, genetic background, season and period of calving, health status, and environmental factors [2]. This coincides with the values published by Hernández et al. [77], Vázquez-Luna et al. [78], and Gutiérrez et al. [79] that found 240–270 days periods. The Italian Mediterranean breed, registered in the Buffalo Genealogical Book, has an average lactation period of 270 days [2]. Once the milk-obtaining process begins, managing the milking program comes to play a crucial role, although it can foster negative behaviors in females triggered by external factors such as the inadequate maintenance of milking machinery and inappropriate practices by operators [23,80,81]. This process requires a series of decisions concerning the conditions of dams and calves, beginning with the choice between manual and mechanical milking (Figure 8 and Figure 9) and possible modifications in the movement or location of animals in certain areas (Figure 7). Breeders must also decide between applying exogenous oxytocin to the females during mechanical milking or using their calves to provide the somatosensory stimulation required for milk ejection.

**Figure 10:**
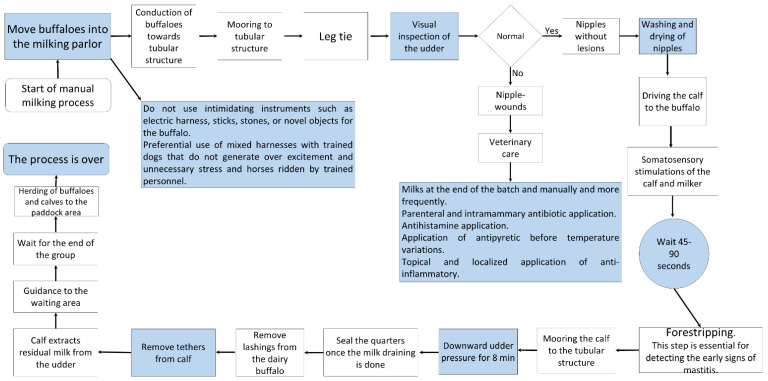
Flow chart of the characteristics of manual milking. In the dual-purpose production system, the presence of clinical or subclinical mastitis is low; however, if it occurs, veterinary care is necessary.

Because most of the milk of the female buffalo is stored in the alveolar compartment (95%), it can be ejected through the action of oxytocin on myoepithelial cells [9,82,83]. Milk ejection is induced by a reflex triggered by stimulating the teat that activates dermal receptors which send signals to the spinal cord and hypothalamus for the release of oxytocin into the bloodstream. This reduces the interalveolar pressure through the contraction of the myoepithelial cells that surround the alveolus, allowing the milk to flow towards the cistern of the udder for subsequent ejection. Oxytocin also aids in reestablishing normal mammary blood flow [84,85] (Figure 10). If the calves are not used to stimulate milk ejection during mechanical milking (Figure 8), then exogenous oxytocin must be administered to ensure milk ejection.

On this topic, Murtaza et al. [86] found that 30-UI of exogenous oxytocin in female Nili-Ravi buffaloes increased milk production when compared to the group of untreated dams. Similarly, Espinosa et al. [82] compared milk production using calves and down-stimulation and the application of 20 UI of oxytocin. The highest values found correspond to the latter group.

Though studies of female buffaloes reveal productive advantages of using exogenous oxytocin, Faraz et al. [87] found that daily applications of oxytocin can affect the mineral content of the milk produced. They recommend suspending the regular use of this hormone because of its impact on consumers. Other observations show that daily oxytocin administration affects females’ reproductive indicators, leading to prolonged periods of anestrus due to slow uterine involution [88,89].

## 8. Productivity during Lactation

**Figure 11:**
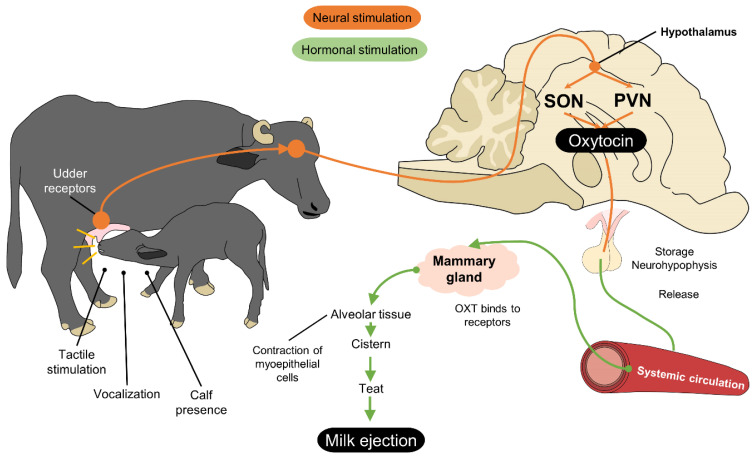
The role of oxytocin (OXT) and suckling behavior. Buffaloes require the presence of the calf and tactile, auditory, and olfactory stimuli for the neural and hormonal stimulation of milk release. The neural pathway begins in receptors located in the udder that are stimulated by the newborn. This stimulus is carried to brain nuclei in the hypothalamus, specifically the SON and PVN, which are responsible for producing OXT. Subsequently, the OXT produced is stored in the neurohypophysis, ready to be secreted into the bloodstream when maternal stimuli are perceived. Once in the mammary gland, OXT binds to specialized receptors in the alveolar tissue, causing contraction of the myoepithelial cells. This allows the milk to pass from the alveolar tissue into the cistern and teat. Finally, milk is ejected in a cycle in which the calf plays a key role essential. PVN: paraventricular nucleus of the hypothalamus; SON: supraoptic nucleus of the hypothalamus.

Studies of the DPBPS show an average milk production of 5.4 L/day during lactation periods that last around 240 days, for a total of 1300 L/cow. It is important to note that a portion of this milk production is destined for calves, since their presence during manual milking constitutes a series of visual, olfactory, and sensory stimuli for the milk ejection (Figure 11), so these values refer exclusively to the milk destined for the market. Reports from Brazil indicate that milk production values range from 1500 to 4500 L per lactation, varying according to the degree of intensification of different production units. It is important to analyze cost–benefit ratios here to verify whether investing in intensive systems is justified.

Productivity levels of lactation are dictated by genetics, environmental, and alimentary factors, management systems, number of lactations, seasonality, and animal welfare. The transition period is especially important due to the impact of the neuroendocrine changes that occur during and after parturition and during lactogenesis on the health, welfare, and productivity of female buffaloes [90,91,92]. Zicarelli [93] concluded that over the past 10 years, milk production by female buffaloes has been fostered worldwide due to the characteristics of these animals allowing them to develop in a resilient and efficient manner, and their capacity to potentiate sustainable production models [94]. This aspect takes on great importance when we consider the high nutritional value of buffalo products, which could respond well to consumption habits associated with good nutrition and health [14].

## 9. The Importance of Allosuckling for Offspring and Dams

As mentioned above, allonursing/allosuckling (communal nursing) refers to the practice in which a female allows (accepts) calves that are not her biological progeny to feed from her udder while she cares for and protects them [95]. This behavior is seldom seen in ungulates, but female buffaloes have shown the capacity to passively accept calves from other biological dams [96], although in a study of 35 female buffaloes with their calves conducted by Mandella-Oliveira et al. [97], it was found that events of nonfilial nursing are not common in this breed after observing daily frequencies of just 0.61–0.67, compared to filial feeding (0.61–1.06).

Several theories have been proposed to explain why these dams allow non-filial calves to suckle and, in the other direction, why calves seek to obtain milk from other females in the herd [62]. Some theories hold that allonursing reflects the female buffalo’s maternal instincts and that factors such as kinship, reciprocity, parental care, social benefits, milk-dumping, and misdirected care may be involved [98,99]. Other proposals center on the newborn’s efforts to satisfy its requirements, emphasizing phenomena such as milk theft, compensation, immunological benefits, and better nutrition [99].

Water buffaloes are seasonal polyestrous animals. Females present estrus most often in the months with the shortest days, which means that births are concentrated in one period of the year, producing groups of newborn calves that might facilitate allosuckling/allonursing. The closeness characteristic of water buffalo herds in terms of the number of animals and the genetic kinship among them is another theory posited to explain why females permit allosuckling by the calves of dams with which they share consanguinity [100]. A study of 34 female buffaloes and 31 calves of Murrah and mixed breeds analyzed the effect of consanguinity on 570 attempts to perform allosuckling, where 351 were successful. In those cases, four to eight calves were observed to feed from non-filial dams, and 13 were involved in the communal care of the newborns. These results suggest that the calves that attempted to feed from females considered their dams’ sisters or half-sisters had less success ($\bar{x}\;$= 0.457) than those that had no kinship ($\bar{x}\;$= 0.563) [95], so that kinship may not be of special importance in this type of behavior in this species.

Something similar occurs regarding the reciprocity theory, which highlights allosuckling as an act of communal rearing [101]. However, other studies report that up to 85% of the buffalo dams that acceded to care for non-filial calves did so regardless of whether or not their own calves had been fed by herd mates [95]. This acceptance of community maternal care is manifested mainly by young or primiparous females, perhaps with the goal of improving their maternal abilities, or due to their inexperience [102]. In support of this, one experiment found that 97% of 30 buffaloes that participated in allonursing were young females with limited maternal experience [95]. In the case of high milk production cows, allonursing has been explained as a way to evacuate the milk that remains after their own calves finish feeding [103]. In this regard, Oliveira et al. [104] found that the females that fed both their own and non-filial calves had greater daily milk production and total milk production than the ones that did not participate in this practice. They further determined that dams with male calves produced larger amounts of milk and were more prone to accepting allosuckling.

With respect to the sex of the newborns, a study of 29 female Murrah-Mediterranean buffaloes recorded that most cases of allosuckling occurred between November and February, during the first 4 months of extra-uterine life, and that they had an average maximum duration of approximately 7 min/day. In another finding, male calves attempted to feed from non-filial dams more often than females (1.52 vs. 1.37 times per day). Their results led those authors to suggest that allonursing in buffaloes occurs as a way to (i) increase fitness in the herd, and (ii) ensure the survival of newborns [105].

Another advantage that calves may obtain with allosuckling is improved nutrition. In this approach, the calf’s sex and birth order are factors that have been related to the incidence of allosuckling and that influence weight gain. A study by da Costa et al. [106] found that male calves had higher weights after longer periods of care, and calves born towards the end of the season had to compete harder to feed from their own dams because they had older calves. Those authors recommend separating young animals into age groups, and having handlers identify the females that are prone to allonursing to safeguard the growth and development of all calves. Not surprisingly, the calves of dams with low milk production are most prone to suckling from other females [95]. It seems clear that calves learn to identify the dams that accept both their own calves and non-filial offspring and tend to approach them for milk [107]. It is important to keep in mind that allosuckling may provide immunological benefits to the calf if it obtains a broader range of antibodies and immunoglobulins (IgA, IgG) [108], though it may entail a risk of infection for the dam (e.g., paratuberculosis) [23,109].

The currently available evidence indicates that water buffaloes, unlike dairy cattle selected specifically for production, do not let down milk in a conditioned manner when stimuli such as milking pails or machines are present [110]. Rather, they need their own calf to provide the visual, olfactory, and auditory stimulation required to generate the neurohormonal control of milk release by oxytocin [95]. For this reason, milk theft is one of the theories most often proposed for species such as buffaloes that have only one offspring at a time, and for which providing milk to non-filial calves entails enormous energy expenditures without bringing any benefits for the dam or her own newborn [111].

**Figure 12:**
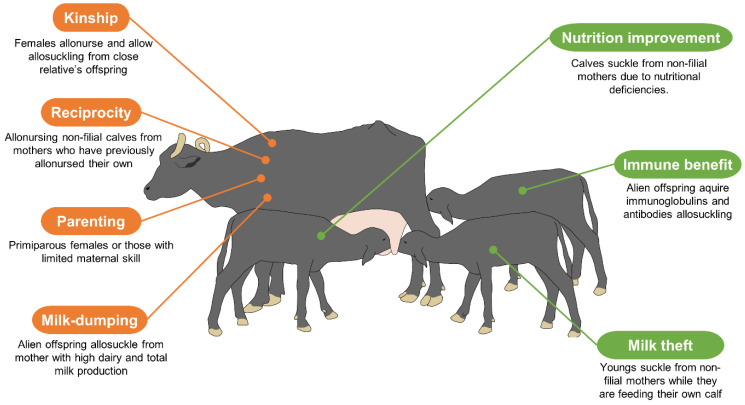
Main hypotheses regarding allosuckling in water buffaloes (*Bubalus bubalis*).

Interested in allonursing, Napolitano et al. [112] evaluated water buffalo calves whose dams did not produce sufficient milk, considering that those newborns suffered hunger. It was found that these kinds of calves were motivated to steal milk from other females to compensate their lack of food (the basis of another theory) [105]. Allonursing in general is less common in animals that bear only one offspring because it is easier for those females to identify an alien calf [101]. In water buffaloes, allonursing is usually seen while the dam is feeding her own calf because at that time a non-filial calf may be able to approach and suckle to disguise its presence [113]. As a result of these studies, allonursing in water buffaloes is not thought to constitute a factor of natural or artificial selection of great significance [95]. However, when it happens, it may have benefits for both the dam and the newborn, such as increased milk production or better nutrition, though its implementation and the risks involved depend largely on the goals of the production system and the sanitary and innocuity measures applied [99] (Figure 12).

## 10. Confinement Systems and Herding

Since the mid-1990s, the growing human population and its demand for foods have induced the formation of organizations, systems, and groups dedicated to generating strategies to satisfy food needs [114,115]. This has brought about important changes in traditional, extensive livestock production systems by moving them towards more intensive models based on confinement. This has impacted several livestock species, including the water buffalo, because it can increase yields to respond to the growing demand for final products. The process of reconversion towards confinement in buffalo production units involves developing more controlled environments and implementing stricter programs of genetic selection and nutritional enhancement [114]. These changes, however, may have consequences for the welfare of the animals due to greater human–buffalo contact, modified feeding regimes, and restrictions on the amount of space available per animal. Observations in production units that have taken this direction have identified both physiological and behavioral responses [66] that need to be analyzed to quantify their impact [80].

The early calf–dam bond established in this species is essential for the calf’s survival and welfare [9,14,63], and must be respected in confinement-based production systems to prevent the economic losses that will surely occur if this is not done.

Breeders who are conscious of the significance of this bond have developed various housing procedures for calves at weaning, from pasture cells with or without the presence of the dam, to total confinement. The latter strategy requires independent areas inside the production unit to facilitate the movement of buffalo steers using one of various herding techniques available to drive them to the fattening area.

### Mobilization by Mixed Herding

**Figure 13:**
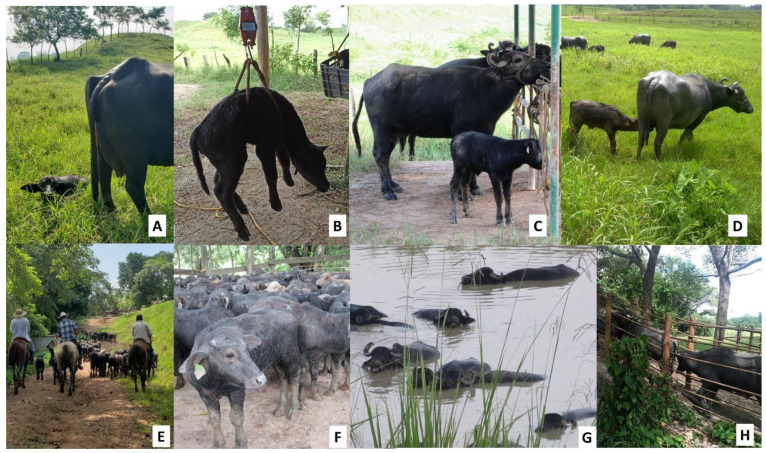
Areas of dual-purpose buffalo production systems. (**A**) Potrero destined for prepartum and delivery days. (**B**) Calf weighing area. (**C**) Manual milking with the presence of calf. (**D**) Grazing in the pasture of the dam in the company of the calf. (**E**). Herding of calves on horseback. (**F**) Confinement of calves after grazing with the dam. (**G**) Specific paddocks for steers where the characteristics that allow their natural behavior of thermoregulation are provided. (**H**) Loading of finished animals or intermediate fattening.

The movement of calves inside production units can require various techniques and may or may not include the use of physical tools or auditory equipment to induce movement. These methods include one called mixed herding, which involves personnel on horseback and dogs [116]. This form of herding tends to be used when travel distances are great and/or using motorized transport is not feasible. In Mexico’s tropical wetlands, mixed herding is often employed in buffalo production systems to move females and their calves, after birth, to different areas for milking or confinement, or into paddocks. In the latter, the animals are moved to areas of pastureland (called paddocks) until weaning. Weaning usually occurs when calves reach a weight of approximately 240–270 kg (Figure 13).

In extensive or semi-extensive systems, moving the buffaloes in different stages of their development is essential. Experience shows that herding methods influence daily milk production and have repercussions on the weight gain of calves as a consequence of stressful factors such as handling and the aggressive, even painful, use of tools [117].

Some studies report that using dogs is another source of stress during operations that constitute poor animal handling [117,118,119]. Kuhl [120] described the use of dogs and horses as a critical point that provoked stress in the animals as they moved, and caused injuries due to poor training in their use. Huertas [121], in turn, recommended not using instruments—or unknown, poorly trained handlers—to force buffaloes to move in order to prevent threats, nervousness, and excitation.

Observations in Mexico show that animals herded by handlers on horseback presented lower cortisol levels, which meant a 15.3-times lower likelihood of producing tough, dark, dry meat, compared to the animals that were herded by handlers on foot. This was attributed to the time required in each case, in part because the buffaloes put up less resistance when herded by mounted handlers. Another factor identified was the effect of poorly trained personnel whose actions affected the welfare of the animal and triggered more acute states of alert [122]. Mixed herding, therefore, is functional and suitable, and is likely one of the best options for moving animals and for displacements over long distances, where goals include preventing stress, fear, and any deterioration of animal welfare during such mobilizations.

## 11. Handling during the Loading, Transport, and Unloading of Buffalo

Turning to the male buffalos, most of these animals are sold after weaning or at the intermediate fattening stage, depending on the availability of forage at each production unit and prices in markets: if prices are low and good forage is available, the animals may be retained to gain weight while awaiting price rebounds that will benefit producers. This is feasible thanks to the flexibility that distinguishes dual-purpose production systems [25].

After weaning, the next stage of production, often called intermediate-fattening, stage involves transporting weaned animals weighing about 270 kg, and/or aged about one year, to specific finishing areas. This stage is usually carried out at a distinct location, so the buffaloes are subjected to handling activities such as loading, transport, and unloading. These management practices require herding and mobilization techniques and procedures that involve vehicles and unloading in the fattening area. Before this process begins, the animals are housed in paddocks. As in the case of the calves and female milk producers described above, steers are also normally herded using the mixed method that facilitates their movement over middle to long distances, thus avoiding both loss of time and lesions caused using foreign objects. After herding, the animals enter a lairage corral or a tubular-shaped handling chute in single file and enter the transport vehicle by climbing a stationary concrete ramp designed to facilitate their ascent. Upon arrival at the fattening and finishing area, similar processes are followed to unload the animals down through stationary ramps that connect to pathways to the reception corrals. Finally, they are released into new sites, often characterized by pasturing systems or semi-extensive feedlots with areas of confinement to shorten finishing times. The exact conditions vary from one production unit to another.

The animals destined for meat rather than milk production are transported to a different site at least once during their lifetime [123]. Currently, buffaloes can be transported by railroad, ship, airplane, or truck to areas dedicated to fattening, breeding, or slaughter, but available evidence suggests that confinement in a moving vehicle is a stressful event for these animals [124]. Especially important here are issues such as the amount of space allowed per animal, load density, driving styles, road conditions, vibrations, strange sounds, and prolonged trip times that can result in lesions, damaged meat in the canal, and deteriorated meat quality, among other problems [116,125,126,127].

For these reasons, transport is closely associated with reductions in the animals’ immune system, the impact of which is mediated, preferentially, by the hypothalamic-adrenocortical axis and the consequent secretion of corticosteroid hormones [128]. Associated disorders were studied by El-Deeb and El-Bahr [129] in a group of 50 water buffalo calves divided into two groups. One group was subjected to transport, while the other was not. Hematological and biochemical parameters were analyzed after unloading post-transport. Their results show increases in the serum concentrations of acute stage proteins—haptoglobin, serum amyloid A, and fibrinogen—in the transported calves [129].

While precise transport conditions can vary depending on producers’ objectives, transporting an animal without companions from its group can generate signs of stress. For this reason, the recommendation is to transport animals with a congener or, if this is not feasible, implement alternatives that reduce the isolation effect, such as placing mirrors inside the vehicle [128]. The transport of water buffaloes in the study area is usually by trucks equipped with tubular structures that can carry up to 60 animals, or smaller vehicles adapted to carry smaller loads of just 1–3 animals per trip.

There is scarce information on the incidence of lesions and economic affectations caused by the transport of buffaloes, but in Bangladesh, Alam et al. [126] studied a sample of 560 *Bos* genus bovines and water buffaloes. They determined that 89% of all the study animals had at least one lesion, but that 99% of the buffaloes presented evident cutaneous lesions, compared to 84% of the cattle [126].

Following the same scheme, scores for hematomas in cattle transported at high densities (0.89 m^2^/animal) have shown a strong relation when compared to animals transported at medium loading densities (1.16 m^2^/animal). Results such as these reveal the need to evaluate—and determine—suitable loading densities in accordance with species, the groups of animals to be transported (i.e., calves, feeders, finished cattle, culls, and so on), the characteristics of the vehicles, and the prevailing climatic conditions during transport [123].

Another study assessed tail and muzzle lesions in buffaloes and *Bos* genus bovines during handling. The authors determined that 54% of the water buffaloes suffered injuries due to the tearing or chafing of the nostrils caused by the use of ropes looped through perforations [130]. Tail lesions were found in 39% of the *Bos* genus bovines and buffaloes. The predominant injury (98%) was bent tails [130].

The unloading process used depends on the characteristics and infrastructure in the reception area of the production unit, but it is common to observe similar facilities, consisting of stationary concrete ramps and tubular chutes.

## 12. Handling, Processing, and Commercialization of Milk

In Mexico, few water buffalo products are fully differentiated from other products due to the absence of established norms to promote schemes of product tracing and the lack of valorization products and their derivatives. In the case of milk, current selling prices range from 9 to 10 peso/L, around 20% higher than the price of bovine milk. This is because buyers value it for its high yields in the elaboration of cheeses and other byproducts.

While water buffalo producers in Mexico consider this activity profitable, they know that this depends on three principal factors: daily weight gain, liters produced per lactation, and the number of calves born per year. Obviously, the production costs of milk, meat, and derived dairy products such as cheese, among others, are fundamental in determining the levels of profitability of production units. In this regard, production in pasture systems is essential because of its advantages for reducing production costs [78].

The commercialization of water buffalo products has increased gradually in Mexico, but obstacles to distribution and access to more demanding markets continue to exist because their quality, hygiene, safety, and nutritional value are not guaranteed. Due to these circumstances, the commercial channels available to producers are mostly popular markets. There, sanitary controls are less strict and transactions are dominated by intermediaries and regional-level processors that tend to offer prices lower than those of industrial and organic markets [10,131].

There are, however, countries that have a broad development of products made from buffalo milk that have high added value. In Italy, for example, the market demand for and the value of buffalo milk are higher, sometimes tripling the selling price of products of *Bos* genus origin, because of its high value for mozzarella cheese production. Studies show that the yield in cheese elaborated with milk from Mediterranean buffaloes is 25.5%, compared to just 12.5% for dairy cattle [93].

Circumstances in Bangladesh are quite unique, as producers raise the Murrah, Nili-Ravi, Surti, and Jafarabadi breeds, constituting an important economic, social, and cultural resource, even though these are considered the least favorable breeds for milk production due to the poor practices applied and low prices for milk, worsened by the fact that byproducts are usually distributed through informal commercial channels [90,132].

To stimulate the economy of water buffalo producers, it is necessary to value the benefits of water buffalo milk. This will require the opening and recognition of a differentiated market where strategies can be applied combining productive aspects (including measures to ensure hygiene and innocuity) with marketing initiatives to inform and convince consumers. In the degree that this is implemented, it will be possible to follow the route traced in Italy by publicizing the properties and benefits of mozzarella cheese made from water buffalo milk to increase sales. To give one example, exports of mozzarella cheese to Japan, South Korea, and the United States increased by 16.4% between 2016 and 2017 [133]. This case highlights the potential that exists, but also the need to induce changes in the management of productions units and the chain of commercialization of water buffalo products.

## 13. Conclusions

The development of dual-purpose water buffalo production systems in Mexico’s tropical wetlands is a relatively recent phenomenon that has progressed and improved due to herd management that combines the accumulated experience of ranchers with innovations generated at the national and, above all, international levels. However, as this review shows, there are many areas that need to be addressed for improvement, including the abandonment of offspring, routine practices in animal management, and a deep understanding of behavioral and physiological patterns in water buffalo, among others. Buffaloes are an interesting alternative for dual purpose systems that offer several advantages: They move in groups, facilitating their adaptation to rotating pasture systems. Their rusticity makes them feasible to exploit flood-prone areas and fields with low-to-medium quality grasses. Moreover, water buffaloes in Mexico have shown a significant resistance to many of the plagues and diseases that severely affect conventional cattle.

Ranchers are gradually adopting better buffalo reproductive practices. Advances in artificial insemination have been made in Mexico, but it is still difficult to obtain high-quality semen or sexed semen. Embryo transfer is not practiced due to the lack of the material required in the region.

Colostrum and cow–calf relationships are highly valued, as ranchers understand that the presence of the calf and its consumption of part of the milk produced by its dam stimulate milk ejection and represents health benefits for both. However, the introduction of mechanical milking in some production units has modified this routine and the calf’s role has been taken over by the administration of oxytocin, though this change affects the dam–calf bond and the full exploitation of high-quality colostrum.

In contrast, some producers have begun to exploit allonursing during calf-rearing, though it is not clear how the best use could be made of this practice.

The lower milk production of this species compared to cattle is its main limitation. However, the properties of buffalo milk allow one to obtain an added value and make this type of farms competitive. This, however, could be potentiated by establishing specific norms for buffalo milk including commercial differentiation and campaigns designed to inform consumers by emphasizing the value and quality of buffalo milk, meat, and byproducts.

In synthesis, consolidating buffalo production in Mexico’s tropical wetlands will require broadening our knowledge of this species, and perfecting the most appropriate handling procedures. Only in this way will it be possible to raise the efficiency of the dual-purpose production system and improve the standard of living of primary producers. The activities of government agencies and processing enterprises will play vital roles in achieving the integral modernization of this potentially important economic activity.

## Data Availability

Not applicable.
